# Polymerase/DNA interactions and enzymatic activity: multi-parameter analysis with electro-switchable biosurfaces

**DOI:** 10.1038/srep12066

**Published:** 2015-07-15

**Authors:** Andreas Langer, Michael Schräml, Ralf Strasser, Herwin Daub, Thomas Myers, Dieter Heindl, Ulrich Rant

**Affiliations:** 1 Dynamic Biosensors GmbH, Lochhamer Str. 15, 82152 Martinsried, Germany; 2 Technische Universität München, 85748 Garching, Germany; 3 Roche Diagnostics GmbH, Nonnenwald 2, 82377 Penzberg, Germany; 4 Roche Molecular Systems Inc., 4300 Hacienda Dr Pleasanton, CA 94588, USA

## Abstract

The engineering of high-performance enzymes for future sequencing and PCR technologies as well as the development of many anticancer drugs requires a detailed analysis of DNA/RNA synthesis processes. However, due to the complex molecular interplay involved, real-time methodologies have not been available to obtain comprehensive information on both binding parameters and enzymatic activities. Here we introduce a chip-based method to investigate polymerases and their interactions with nucleic acids, which employs an electrical actuation of DNA templates on microelectrodes. Two measurement modes track both the dynamics of the induced switching process and the DNA extension simultaneously to quantitate binding kinetics, dissociation constants and thermodynamic energies. The high sensitivity of the method reveals previously unidentified tight binding states for *Taq* and Pol I (KF) DNA polymerases. Furthermore, the incorporation of label-free nucleotides can be followed in real-time and changes in the DNA polymerase conformation (finger closing) during enzymatic activity are observable.

The DNA polymerases are the workhorses for replication of genomic information in living cells but more recently also for biotechnology applications such as the polymerase chain reaction (PCR) and DNA sequencing. In particular in the field of sequencing-by-synthesis (e.g. nanopore sequencing), massive efforts are currently being undertaken to develop working principles for faster, more accurate and consequently more cost-effective assays^1^,^2^, designed ultimately, to operate at the single molecule level in medical applications. To this end, high-performance polymerases must be carefully engineered^3^,^4^, an approach which relies on having precise knowledge about their mode of action^5^.

Figure 1 illustrates a simplified DNA polymerization process (see reviews^6^,^7^,^8^,^9^ for more details): first, the DNA polymerase (P) binds to the junction of an oligonucleotide primer hybridized to a complementary oligonucleotide template DNA with high affinity (1 → 2). Second, the P-DNA complex binds a deoxynucleoside triphosphate (dNTP) with comparably weak affinity (2 → 3), which depends on the type of adjacent base on the opposing single stranded template (base-pairing), so that a complementary dNTP is favored. Third, the polymerase incorporates the nucleotide by catalyzing the formation of a phosphodiester bond to the 3’ end of the primer, thereby extending the strand by one nucleotide and producing pyrophosphate (PP_i_) waste (3 → 7). In more detail, the catalytic action involves several steps, where the polymerase conformation plays a crucial role: the dNTP binds to the P-DNA complex when the finger domain is in an open state (3); thereafter, the fingers close P_O_ → P_C_ (4) and the dNTP is aligned with the enzymatically active site, where the chemical step takes place and the nucleotide becomes incorporated (5). After the finger domain has opened again (6), PP_i_ product is released and the polymerase translocates forward to the next nucleotide in the template strand. For mismatched dNTPs, the fingers only partially close P_O_ → P_PC_ (4)^10^,^11^, which alters the probabilities of incorporation and dissociation and effectively constitutes a fidelity check that favors the incorporation of correctly paired bases.

Despite its very simplified nature, the scheme in Fig. 1 is practical because it represents the elongation process as the two steps of Michaelis-Menten-like enzyme kinetics, which involves a minimal set of *interaction* and *activity* parameters for the assessment of the polymerization process. The formation and stability of binary and ternary complexes can be predicted from the kinetic rates (*k*_*on*_, *k*_*off*_) and the dissociation constants (*K*_*D*_) of polymerase, template DNA, and dNTPs, respectively. Together with the enzymatic parameters *k*_*cat*_ (turnover number) and the Michaelis constant *K*_*M*_ = (*k*_*off*_ + *k*_*cat*_)/*k*_*on*_ (substrate concentration of half-maximal conversion velocity), the performances of different polymerases can be compared.

However, the reaction pathway of Fig. 1 imposes significant challenges on the measurement modalities employed to resolve the individual steps and unravel interdependencies: The involvement of many interactants (P, template DNA, competing DNA, matching/mismatching dNTPs, Mg^2+^, protein cofactors, PP_i_), gives rise to a number of combinatorial situations that are difficult to manage experimentally, especially when DNA sequence variations are of interest. Moreover, different interactions take effect at distinct concentrations spanning a wide range from *pM* (P-DNA) to *mM* (P-Mg^2+^), which requires a highly sensitive measurement system with a large dynamic range. In order to assess the functionality and yield of the polymerization process, it must be possible to monitor the DNA elongation in real-time and to detect transient changes in the polymerase conformation. To date, no single assay method has been able to meet all these criteria.

Chemical-quench methods with radiolabeled molecules were introduced in the 1970’s and were widely used to provide kinetic data, with methods further improved with the introduction of automated stopped-flow instruments. These instruments enabled the rapid mixing of solutions^9^,^12^,^13^,^14^,^15^,^16^ but the handling of radioactive substances remained difficult and laborious. The use of fluorescence labels is advantageous for many purposes^17^,^18^,^19^,^20^,^21^,^22^,^23^,^24^ but does not allow the quantification of high-affinity interactions with *pM* 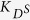 in solution, because the labelled compound must be present at *nM* concentrations to enable the detection with fluorescence (anisotropy) spectrometers^24^,^25^,^26^,^27^. Additionally, the label may severely interfere with binding sites and enzymatic activity; for instance, dyes inevitably suppress the incorporation of nucleotides, irrespective of whether the nucleotide is directly labelled^19^ or DNA intercalating dyes are being used^21^. Single molecule fluorescence resonance energy transfer (FRET) was successfully used to monitor elongation^28^, nucleotide selection^29^, and conformational transitions of the finger domain of the Klenow fragment^11^,^30^ that had been predicted from crystallography^10^. Since FRET studies rely on elaborate strategies for site-specific labelling^30^, their general applicability remains however limited. Driven by the quest for new sequencing technologies, valuable insight into polymerase activity has also been obtained in single-molecule studies with nanopores^31^,^32^.

Biosensors, where one interactant is immobilized on a surface, are in many ways better suited than solution methods for the dissection of individual pathways in complex reaction schemes. Interactants can not only be provided but also be withdrawn from the reaction using microfluidic channels for solution exchange with association as well as dissociation phases monitored in real-time. Many combinations of interactants at different concentrations can be tested in one assay which allows for the analysis of more complex reactions, speeds up the workflow and makes interpretations more straightforward. Kinetic measurements on P-DNA interactions on surfaces have primarily been performed with SPR (surface plasmon resonance) sensors^19^,^33^,^34^,^35^,^36^,^37^ and, less frequently, with fluorescence and fiber optics methods^18^,^38^. Some of these methods also have observed the elongation of templates^19^,^33^,^36^,^38^. However, several influences can lead to artefacts and must be carefully considered in the interpretation of surface assays, among them being mass-transport limitations^36^, non-specific binding to surface matrix layers^33^, or crowding and rebinding effects due to high immobilization densities^19^. Most importantly, it is not possible to draw information about the position of the polymerase along the DNA or its conformation (fingers closing transition) from state-of-the-art measurements.

Here we report a method that utilizes a stimuli-responsive molecular interface for the investigation of polymerization processes and does not require the labelling of nucleotides or polymerases. The functional elements are oligonucleotide probes which are assembled at a very low density on gold microelectrodes. By applying alternating electrical potentials these probes are set in motion and perform an oscillatory orientation switching from which two types of measurement variables are obtained in real-time: The switching speed depends on the hydrodynamic friction of the probes and thus indicates the presence of a bound polymerase, its position along the DNA, and its conformation. At the same time, the extension of electrically aligned “standing” DNA molecules is measured, revealing how many base-pairs have formed in the course of polymerization activity. From these two complementary signals a number of parameters characteristic for the interaction (affinities, kinetics, thermodynamic energies) and the enzymatic activity (elongation rate, Michaelis-constant, changes in the polymerase conformation) can be analyzed with unprecedented sensitivity. This is demonstrated for the *Taq* DNA polymerase from *T. Aquaticus* and the Klenow fragment of Pol I from *E. coli*. The described assay is applicable to DNA and RNA polymerases and is performed using commercially available chips and instruments.

## Results

### Measurement Principles

Figure 2 illustrates the two complementary measurement principles, which derive information about the orientation/extension of surface-tethered oligonucleotides from the quenching of a fluorescent dye in the proximity of a metal film. Experiments are performed with DNA layers comprising app. 10^6^ strands on microelectrodes with diameters of 120 μm. The optically excited Cy3 dye transfers energy by near-field interaction to the gold film over long distances (>100 nm)^39^ and by measuring the emitted fluorescence intensity, the height of the top end of the DNA can be determined.

The first measurement principle, the dynamic measurement mode, involves an electrical actuation of the DNA by applying alternating voltages of typically ±0.4 V to the gold microelectrodes (Fig. 2A). The negatively charged DNA is repelled from the negatively charged surface and then attracted to the positively charged surface^39^, and oscillates (switches) between lying and standing orientations at typically 10 kHz frequency. This movement is monitored in real-time by time-correlated single photon counting, which generates a fluorescence histogram every second to resolve the upward and downward motions as well as the steady state fluorescence levels of lying and standing DNA, respectively (switchSENSE technique)^40^.

Figure 2B compares upward switching traces of 54-mer DNAs before and after binding the *Taq* polymerase, and before and after the incorporation of dNMPs by the polymerase. Throughout this work a random sequence of a 36 nucleotide (nt) primer hybridized to a complementary 54 nt template, denoted 36/54 or ss-dsDNA, and its fully double stranded analogue, denoted 54/54 or dsDNA, are used. The association of *Taq* polymerase slows the motion of the DNA strand by increasing its hydrodynamic drag, and thus produces a pronounced shift (tilt) in the time-resolved upward switching curve. Upon addition of dNTPs, the curve tilts even more, which suggests that the polymerase elongates the primer and moves up along the DNA (solutions to the drift-diffusion equation^41^ imply that a polymerase located at the distal end of a DNA nanolever indeed features a higher rotational friction coefficient than a polymerase located closer to the pivot point). The polymerase can be removed by flowing a strong denaturation agent (5 M urea) above the surface; thereafter, the upward switching trace coincides with the control measurement of a 54/54 layer, which corroborates the notion that the polymerization from 36/54 to 54/54 was successful.

For ease of analysis, the time-resolved upward switching curves are converted to a single value, the Dynamic Response parameter (*DR*_up_), which is the area under the normalized fluorescence curve:


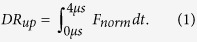


The *DR*_*up*_ represents the switching speed: high *DR*-values indicate fast switching, low *DR*-values indicate slow switching.

A second signal provides valuable complementary information on the polymerase activity: the absolute fluorescence intensity measured for a standing DNA orientation (Fig. 2C,D). In order to align the DNA upright, it is essential to apply a repulsive (negative) surface potential^39^,^41^, since otherwise the DNA orientation is ill-defined and quantitative comparisons cannot be made.

Upon addition of dNTPs to complexes of *Taq* bound to ss-dsDNA, the fluorescence increases significantly. As the polymerase converts the upper DNA part from a floppy single- to a rigid double-strand, the dye effectively moves away from the fluorescence-quenching surface. This is a consequence of the short-ranged electric field that decays rapidly above the surface due to Debye screening^39^,^41^. It does not significantly affect flexible single-stranded DNA segments which are more than a few nanometers away from the surface and thus they can move about freely. By contrast, for a fully double-stranded helix the repulsion of its surface-proximal segment is effectively transduced to the upper DNA end, because the molecule is rod-like. The fluorescence after removing the *Taq* by urea from the polymerized strand is comparable to the fluorescence of the same layer after removing the polymerized strand and re-hybridizing it with a full length 54 nt cDNA.

### Kinetics of the *Taq*-DNA interaction

**Figure 1:**
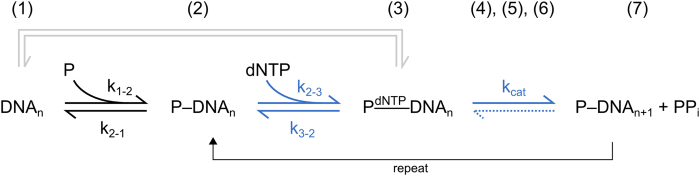
Simplified DNA polymerization reaction pathway polymerase binding (1 → 2), nucleotide binding (2 → 3), nucleotide incorporation (3 → 7). Changes in the polymerase conformation (fingers closing/opening) and PP_i_ release are combined in states (4–6) here, see text. Michaelis-Menten-like steps of substrate binding and product catalysis are indicated in blue. Note that the sequential association/dissociation of binary complexes (1 ↔ 2 ↔ 3) is fundamentally different from the association/dissociation of a ternary complex with all reactants present simultaneously (1 ↔ 3, gray pathway). Influences of cofactors, exonuclease activity and other possible transitions are omitted.

**Figure 2:**
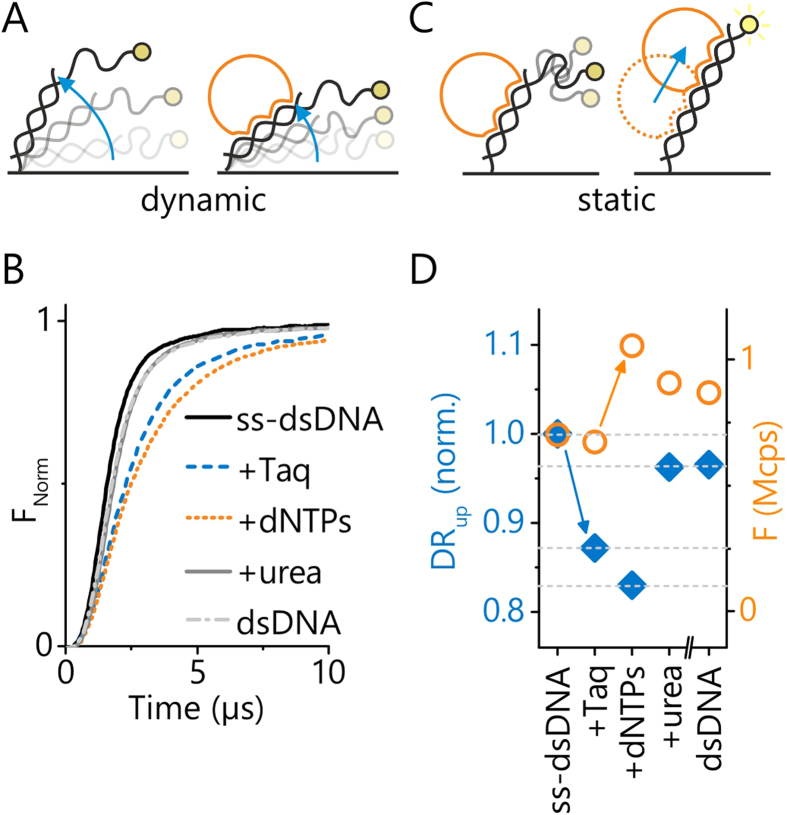
Measurement principles. Both measurement modes utilize the quenching of a fluorescent dye in the proximity of a metal film for evaluation of the distance of the DNA’s top end from the surface in real-time. **A** Polymerase detection by dynamic DNA switching in alternating electric fields: the binding of a polymerase slows the electrically driven DNA orientation-switching due to an increase in hydrodynamic drag (only the upward motion, induced by the positive-to-negative potential step, is shown). **B** Upward switching traces: fluorescence intensity measured by time-correlated single-photon counting during the first 10 μs after switching the electrode potential from attractive (+0.4 V) to repulsive (−0.4 V). **C** Polymerase activity measurement at static DNA orientation: when holding the DNA in an upright orientation by applying a constant repulsive potential, the incorporation of dNMPs results in a fluorescence enhancement proportional to the length of the DNA extension. **D** Comparison of switching speed (Dynamic Response upward, *DR*_*up*_) and steady-state fluorescence during polymerase binding, primer extension, polymerase removal by urea, and removal of the extended primer followed by hybridization with a 54-nt oligonucleotide (control).

**Figure 3:**
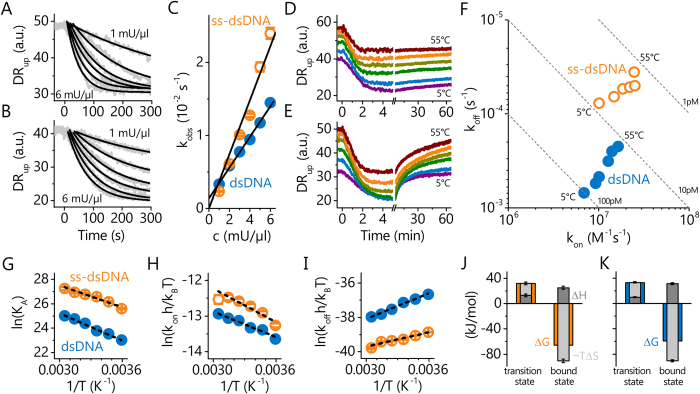
Kinetic and thermodynamic analysis of *Taq* interacting with ss-dsDNA (36 nt primer hybridized to complementary 54 nt template) and dsDNA (54 bp). **A**,**B**: Association of *Taq* to ss-dsDNA (**A**) and dsDNA (**B**) monitored as changes in the Dynamic Response parameter for different *Taq* concentrations (1, 2, 3, 4, 5, 6 mU/μl, U = activity units). Lines are exponential fits, *DR*_*up*_ = *a* + *b*·*exp*{−*k*_*obs*_*t*}. **C**: Rate constants *k*_*obs*_ from **A** & **B** for ss-dsDNA (open symbols) and dsDNA (full symbols) versus *Taq* concentration; solid lines are linear fits, *k*_*obs*_ = *c*·*k*_*on*_ + *k*_*off*_. **D**, **E**: Association ([*Taq*] = 5 mU/μl) and dissociation curves for ss-dsDNA (**D**) and dsDNA (**E**) at 5°, 15°, 25°, 35°, 45°, and 55 °C. F: Map of association- and dissociation-rate constants *k*_*on*_ and *k*_*off*_ from D&E (for dsDNA 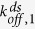 is shown). **G**–**I**: Analysis of thermodynamic energies from van’t Hoff (**G**) and Eyring plots (**H**,**I**) for ss-dsDNA and dsDNA. Lines are linear regression analyses. **J, K**: Energy diagrams for ss-dsDNA (**J**) and dsDNA (**K**) for *T* = 25 °C, shown are the transition and bound states, respectively. Measurements were conducted at pH 8.3 (10 mM Tris, 40 mM KCl, 1.5 mM MgCl_2_).

To follow the association of *Taq* to ss-dsDNA (Fig. 3A) and dsDNA (Fig. 3B) in real-time we monitored the switching speed, i.e. the Dynamic Response signal. It is important to note here that the experiments were conducted in the absence of dNTPs or other DNA in the solution (cf. 1 ↔ 2 in Fig. 1), since this has a profound influence, as will be shown later. The association kinetics can be described well by single exponential fits,


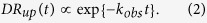


Plotting the observed on-rates *k*_*obs*_ versus the *Taq* concentration in Fig. 3C shows the expected linear relationship


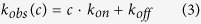


and enables us to determine the intrinsic association rate *k*_*on*_ = *k*_1_ → _2_ (cf. Fig. 1) by linear regression. The on-rate at 25 °C is slightly higher for ss-dsDNA, 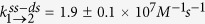 vs. 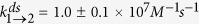, albeit this difference seems small compared to the substantial dissimilarities observed in the dissociation behavior. The non-zero intercept of the linear fit for dsDNA in Fig. 3C indicates that the off-rate is quite significant for continuous double-strands. This is confirmed by real-time dissociation measurements in running buffer without *Taq* (Fig. 3D,E), which show that the dissociation from dsDNA proceeds ten times faster than from ss-dsDNA, 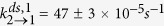 vs. 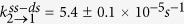 (at 25 °*C*).

In addition to the 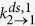-process, the dissociation kinetics from dsDNA exhibit signs of a second dissociation process with an even higher dissociation rate 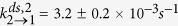, which is not observed for ss-dsDNA. We attribute this to the presence of not only one, but two *Taq* molecules on the 54 bp dsDNA molecules^42^, which feature different dissociation rates. In control experiments with dsDNA that was internally labelled mid-strand, 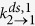 was found to be comparable to dsDNA with a terminal Cy3 dye, but the 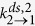-process was not observed (Supporting Information Figure 1). This finding suggests that the 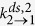-rate stems from weakly bound *Taq* molecules associated to surface-proximal segments of the dsDNA, an interaction which is probably sterically inhibited when an internal Cy3 label is present at the central DNA position. It also confirms that the terminal Cy3 label does not affect the quantitation of the dissociation 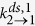-rate.

### Thermodynamics of *Taq*

Association and dissociation measurements were performed at different temperatures from 5° to 55 °*C* (Fig. 3D,E) in order to obtain thermodynamic information about the previously unidentified tight binding state. The corresponding *k*_*off*_ vs. *k*_*on*_ rate map is depicted in Fig. 3F. Generally, the affinity of *Taq* for ss-dsDNA is an order of magnitude higher than for dsDNA. Upon elevating the temperature, the on-rates increase for both DNA types, while, the off-rates decrease. As a consequence, the affinities improve by an order of magnitude from 5° to 55 °*C* and the dissociation constants


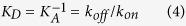


approach very low *pM* values (
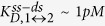
 and 
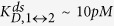
 at 55 °C).

We analyzed the energies of the bound state


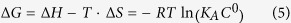


(*C*^0^ = 1 *M* being the standard concentration) from van’t Hoff plots, which are depicted in Fig. 3G. The relationship between ln(*K*_*A*_) and 1/*T* is found to be linear within the measurement accuracy, indicating that the enthalpy and entropy are constant over the investigated temperature range. This behavior is generally expected for non-specific protein-DNA binding where the change in heat capacity usually is insignificant^43^, however, under different experimental conditions heat capacity changes have been observed in calorimetric studies^44^. The negative slope of the van’t Hoff plot signifies an endothermic reaction (Δ*H* > 0), which is outweighed by a strong entropic contribution (−*T*Δ*S* ≪ 0) to give a negative free energy difference (Δ*G* < 0) that drives the reaction towards the bound state. Therefore, the *Taq* polymerase can be considered a purely entropic binder. Interestingly, the entropy gain upon binding is not affected by the presence of a single-stranded overhang, Δ*S*^*ss*−*ds*^≈Δ*S*^*ds*^. The weaker affinity for dsDNA is rather caused by the higher enthalpic penalty (+25%) when binding to a continuous double-strand (Δ*H*^*ds*^ > Δ*H*^*ss*−*ds*^). Energies of the transition state could be analyzed from the available kinetic rate constants using the Eyring equation


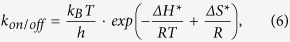


again carrying out linear regression analyses of logarithmic plots, cf. Fig. 3H,I. In this case both, enthalpy and entropy, contribute to the activation energy barrier (ΔH^*^ > 0, −*T*ΔS^*^ > 0 → ΔG^*^ > 0). Energy diagrams are shown in Fig. 3J,K and Table 1 summarizes the activation and free-state energy values.

### Enzymatic Activity of *Taq*

**Figure 4:**
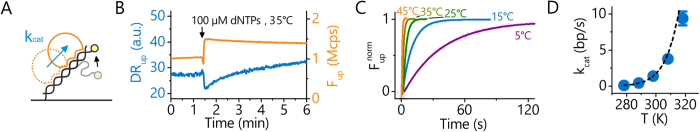
Enzymatic activity measurement of *Taq*. **A**: Schematic of the ss-dsDNA which is converted to dsDNA during dNMP incorporation. As a result, the polymerase moves outward along the DNA nanolever (which increases the friction and slows the DNA switching) and the dye is being held at a greater distance from the surface by the rigid double helix when the DNA is standing (which increases the fluorescence). **B**: *Taq* was bound to ss-dsDNA and baselines are recorded for 1.5 min (no dissociation). The injection of a dNTP-mix (100 μ*M*) leads to a rapid incorporation of nucleotides (*DR*_*up*_-drop and *F*_*up*_-rise), followed by the dissociation of polymerase from the dsDNA end (increasing *DR*_*up*_ and slightly decreasing *F*_*up*_). **C**: Temperature-dependent real-time elongation measurements (*c*_*dNTP*_ = 100 μ*M*). Lines are single exponential fits ∝ *exp*{−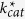
*t*}. **D**: Elongation rates from C as a function of temperature, the line is an Arrhenius fit.

We investigated the enzymatic activity of the *Taq* polymerase during primer elongation by monitoring two signals at the same time, cf. Fig. 4A,B. While the fluorescence intensity emitted by the standing DNA (*F*_*up*_) reports the primer elongation state (height of the upper, Cy3-labelled DNA end), the switching speed (*DR*_*up*_) indicates the polymerase’s binding state and position on the DNA. Upon addition of dNTPs and commencing primer elongation, *F*_*up*_ increases sharply as the Cy3 dye moves away from the quenching surface (cf. Fig. 4B, the transient decrease before the increase results from the passage of an air bubble that is used to separate liquid compartments in the flow system). Simultaneously, *DR*_*up*_ drops because the rotational friction increases as the *Taq* is approaching the upper DNA end. After having reached the DNA’s top end, the polymerase unbinds from the now fully double-stranded DNA and single-exponential dissociation kinetics are observed in the *DR*_*up*_ signal.

The elongation activity of *Taq* was analyzed in real-time as a function of temperature from 5° to 45 °C from the increasing *F*_*up*_ signal in Fig. 4C. The kinetics do not depend on the dNTP concentration, indicating that the activity of the polymerase is the rate-limiting factor (see Supporting Information Figure 2). The elongation curves follow first-order kinetics and can be fitted very well with


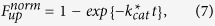


with 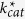 being the elongation rate constant (or velocity) in normalized fluorescence units per second. Since the length of the newly synthesized DNA is known (18 nt), we can convert this rate to units of formed base-pairs per second. For the rather short template used here, elongation velocities up to 10 *bp*/*s* could be followed, but we believe that the analysis of higher velocities should be straightforward when using longer templates. Elongation rates are plotted versus temperature in Fig. 4D and found to triple every 10 °*C*, in agreement with previous results for *Taq*^45^. The line is an Arrhenius fit,


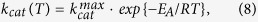


where *E*_*A*_ = 90 ± 4 *kJ*·*mol*^−1^ denotes the activation energy for nucleotide incorporation. This analysis, however, pertains to the case of low temperatures only, since the polymerase becomes deactivated at very high temperatures, which effectively reduces the catalytic activity and requires more elaborate modeling^46^.

### Kinetics and affinities of Pol I(KF)

Pol I (KF) has been the subject of many studies, but the reported binding kinetics and affinities are often inconsistent due to various experimental conditions and due to method-specific influences. In the following we demonstrate how the introduced method can be used to investigate different combinations of interactants in a single workflow.

**Figure 5:**
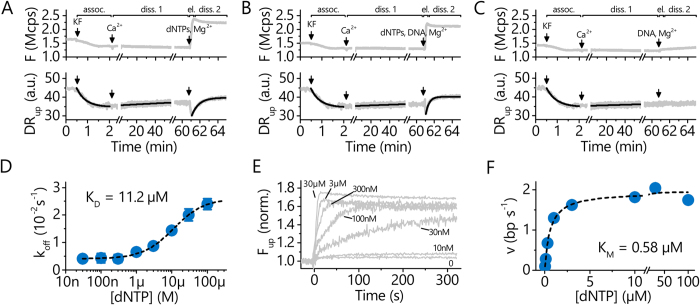
Analysis of P/DNA/dNTP interactions and enzymatic activity of Pol I(KF). **A**–**C** Association, dissociation, and elongation under different conditions: association in Mg^2+^-buffer, followed by dissociation in Ca^2+^-buffer (diss. 1), elongation in a 100 μ*M* dNTP-mix, and dissociation of the polymerase in Mg^2+^-buffer (diss. 2). In A and B, the dissociation phase 2 proceeds after the elongation from the end of the extended oligonucleotide primer in the absence and presence of competing DNA, respectively. C is a control where the polymerase dissociates in the absence of dNTPs but presence of competing DNA in solution. Lines are single exponential fits. **D** Dissociation rates from dissociation phase 2 as a function of dNTP concentration. The line is a Langmuir fit and yields the dissociation constant for the binding of dNTPs by a polymerase located at the end of dsDNA (without the influence of base-pairing). **E**: Elongation monitored in real-time by the fluorescence emitted from standing DNA (*F*_*up*_) for different dNTP concentrations. **F**: Elongation rates from E (linear slope) plotted as a function of the dNTP concentration. The line is a Michaelis-Menten fit with *K*_*M*_ being the Michaelis constant.

At the beginning of each run depicted in Fig. 5A–C, Pol I (KF) was flowed over a ss-dsDNA layer (t = 1–2 min), and the association kinetics were analyzed from the *DR*_*up*_-decrease as before, yielding 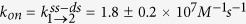. Next, the dissociation of the binary P-DNA complex was measured over one hour, during which the polymerase unbound from the DNA with a remarkably slow rate, 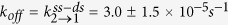. To suppress the 3’-exonuclease activity of the polymerase, the dissociation buffer was Mg^2+^-free, but contained 1.5 mM Ca^2+^ instead to keep the ionic strength constant. The corresponding dissociation constant of the binary P-DNA complex in Ca^2+^-buffer is *K*_*D*,1↔2_ = 1.6 ± 0.8 *pM*. These first two steps were repeated in A-C to demonstrate the reproducibility of the assay.

In a third step, primer elongation was initiated by adding a mix of dNTPs (100 μM) to the binary KF-DNA complex in Mg^2+^ buffer. The rapid incorporation of dNTPs is evident by the increasing fluorescence intensity *F* emitted by the electrically aligned, standing DNA, which saturates within a couple of seconds (Fig. 5A,B). Immediately after the complete double-strand has formed, a fast dissociation process sets in (cf. *DR*_*up*_-increase), suggesting that the polymerase quickly unbinds from the duplex after having reached the end. In the presence of dNTPs and Mg^2+^, but absence of competing DNA in solution (cf. Fig. 5A, phase “diss. 2”) the off-rate is 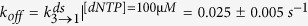 and *K*_*D*,1↔3_ = 1.3 ± 0.4 *nM* (Fig. 5A). The dissociation becomes even faster when competing dsDNA is present in solution (cf. Fig. 5B, phase “diss. 2”), as it is the case for chemical-quench experiments in solution^13^,^47^, 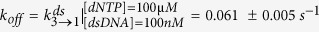 and *K*_*D*,1↔3_ = 3.2 ± 0.6 *nM*.

These values are in agreement with the seminal results of solution measurements from the labs of Benkovic^13^ and Joyce^47^, who used chemical quench methods and radiolabeling to study the KF-DNA interaction in the presence of dNTPs and competing DNA: *k*_*on*_ = 1.2 × 10^7^ *M*^−1^ *s*^−1^ 13, *k*_*off*_ = 0.06 *s*^−1^ 13,47, *K*_*D*_ = 5−8 *nM*^13^,^47^. On-rates (1−3 × 10^5^ *M*^−1^ *s*^−1^) and off-rates (0.002−0.007 *s*^−1^) found in SPR studies^19^,^34^ differ by more than an order of magnitude from solution kinetics and the present results; because the deviations are in opposite directions, however, they compensate in the calculation of *K*_*D*_ values, which are reported to be *nM*, too. The interpretation of SPR data has not been straightforward though and mass transport limitations and rebinding effects due to high DNA densities (cf. 8 × 10^12^ *cm*^−2^ in^34^ vs. <10^10^ *cm*^−2^ here), both resulting in artificially slowed kinetics, have been discussed as adverse influences^19^,^33^,^34^.

We measured the dissociation of Pol I(KF) in Mg^2+^-buffer as a function of the dNTP concentration and obtained a binding isotherm that can be fitted well with a simple 1:1 Langmuir interaction model (Fig. 5D).


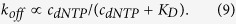


The 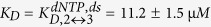 compares well with chemical-quench (6–17 μ*M*)^13^ and FRET measurements (3 μ*M*)^11^ in solutions which were performed in the absence of template DNA. The situation here is similar in some respect: after having polymerized the primer, the KF is still bound to the top end of the now double-stranded DNA, but base-pairing to a template strand is no longer possible. The binding of dNTPs by the polymerase alone obviously induces a fast dissociation of the enzyme from the polymerized strand. It is remarkable, that the affinity of this interaction resembles the affinity of dNTP to free enzyme. Apparently, the fact that the KF still holds on to the dsDNA’s top end does not seem to strongly affect dNTP binding.

### Enzymatic activity of Pol I(KF)

The Klenow fragment of Pol I from *E. coli* exhibits, in contrast to the thermophilic *Taq*, high enzymatic activity at room temperature. We monitored the elongation for different dNTP concentrations in real-time (Fig. 5E) and found that the kinetics strongly depend on *c*_*dNTP*_ and that incorporation proceeds quasi-linear in time. This means that the availability of dNTPs and not the enzymatic activity is the limiting factor for dNMP incorporation by Pol I(KF) at room temperature. The initial elongation velocities *v* were analyzed from the slopes of the fluorescence traces (*dF*_*up*_/*dt at t*≈0), and again converted to units of *bp*·*s*^−1^ taking into account the known synthesis length (18 nt). The *v*-vs.-*c*_*dNTP*_ curve (Fig. 5F) exhibits the typical shape of Michaelis-Menten enzyme kinetics and was analyzed by non-linear regression using the equation


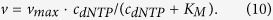


The turnover number *k*_*cat*_ = 2.0 *s*^−1^ (corresponding to *v*_*max*_ in this notation) agrees with solution measurements (2.4 *s*^−1^)^47^. The determined Michaelis constant *K*_*M*_ = 0.58 ± 0.20 μ*M* matches results from primer extension gel measurements (0.41 ± 0.17 μ*M*)^27^. The *K*_*M*_-values found in studies employing fluorescent labels are usually higher (2.8−8 μ*M*)^19^,^21^, which is not unexpected since dyes have been shown to inhibit the binding and hence the incorporation of dNMPs^21^. Notably, solution measurements can also be prone to artificially inflated *K*_*M*_-values, because the amount of added dNTPs must significantly exceed the number of extendable DNA bases, which usually puts the sensitivity of the assay in the μ*M* concentration range. Using a microfluidic channel to provide a constant flow across the biosensor surface such is presented here avoids dNTP depletion even at low dNTP concentrations.

In fact, the *K*_*M*_ value corresponds to the dNTP dissociation constant, since *k*_*cat*_ ≪ *k*_*off*_ ≥ 250 *s*^−1^ 15 and thus


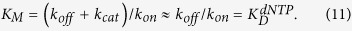


The 0.6 μ*M* value for mixed dNMP incorporation measured here is in good agreement with recent FRET studies reporting 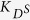 for G-dCTP (0.2 μ*M* and 1.2 μ*M* for the predominant closed and open complex, respectively) and A-dTTP (0.8 μ*M* and 5.1 μ*M*) pairs^11^.

### Detection of the fingers-closing conformational transition

The DNA polymerases are known to undergo a conformational change during enzymatic activity, which facilitates the incorporation of correct nucleotides and helps to reject mismatching dNTPs. The “fingers” subdomain transitions from an open to a closed state, thereby positioning a matching dNTP at the insertion site opposite the complementary template base. The open/closed and even partially-closed conformations have been identified in crystal structures^10^,^48^,^49^,^50^,^51^, and were investigated by FRET measurements in solution^10^,^11^,^30^, but could not be observed with biosensors so far. Since the speed of the induced switching motion depends on the hydrodynamic friction exerted by the polymerase and hence its conformation, we surmised that the fingers-closing transition may be detectable with the current method. To prevent the enzyme from incorporating the dNMP and replicating the template, we used a dideoxy-terminated primer lacking the 3’-OH group which is normally required to join the dNTP *α*-phosphate with the primer backbone. To exclude the influence of exonuclease activity, Pol I(KF exo^−^), with mutations D355A and E357A that abolish 3’ → 5’ exonuclease activity, was used.

**Figure 6:**
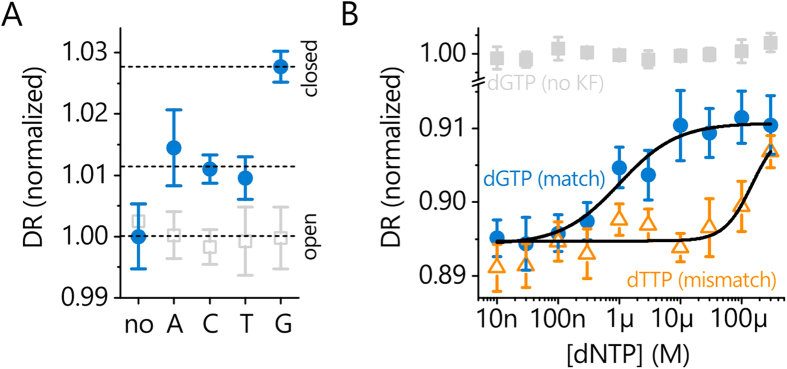
Detection of the fingers-closing transition of Pol I(KF exo^−^) by switching speed measurements. **A**: Incubation of ss-dsDNA with a C at the templating position with 100 μM dNTPs (blue circles) leads to an increase in the Dynamic Response (DR) which is most pronounced for the correctly paired dGTP, indicating a compaction of the protein (fingers-closing). The primer was dideoxy-terminated to prevent the chemical step. Open gray squares are controls without Pol I(KF exo^−^). **B**: Titration analysis of matching (dGTP, circles) and mismatching (dTTP, triangles) nucleotides. Solid lines are Langmuir fits: 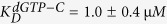, 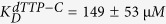. Gray squares are negative control measurements without Pol I(KF exo^−^).

Using this method, a clearly measurable change (+2.8%) was observed in the switching speed upon addition of the correct nucleotide (Fig. 6A). The acceleration of the switching dynamics indicate a compaction of the polymerase conformation upon dNTP binding, in line with expectations for closed fingers, i.e. when the O-helix of the finger domain swings towards the thumb and encloses the dNTP at the insertion site^10^,^11^. Notably, the influence of the finger movement on the overall dimensions of the protein is rather small. Based on the crystal structures of a Pol I(KF) homolog (Bst, PDB accession codes 1L3U and 1LV5 for open and closed fingers, respectively)^10^,^51^, the radius of gyration and protein volume decrease merely from 2.69 to 2.64 *nm* (−2.5%) and 87.7 to 83.4 *nm*^3^ (−5%), as calculated with HydroPro^52^. In spite of this, the switching dynamics measurement successfully resolved the subtle conformational differences, corroborating the high sensitivity of the method.

It is also possible to infer the dNTP dissociation constant from the conformation change by measuring the switching speed as a function of *c*_*dNTP*_. The titration curve follows a 1:1 Langmuir isotherm and the *K*_*D*_ for a matching dNTP was determined at 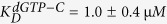 (Fig. 6B), in fair agreement with the value obtained above for a dNTP-mix from extension kinetics. A considerably weaker affinity is found for a mismatching dNTP, 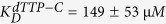 (Fig. 6B). Hence, the discrimination between matched and mismatched dNTPs is more than 100-fold, similar to recent FRET reports from the Kapanidis group^11^. Interestingly, a polymerase compaction is also observable for mismatched dNTPs in Fig. 6A (DR increase by +1%), however, more measurements are required to determine whether this reflects a partially closed (ajar) conformation^10^,^11^, or if the increase is simply caused by the very high dNTP concentration (100 μM) used in Fig. 6A (cf. the onset of the mismatch curve in Fig. 6B).

## Discussion

We have shown that monitoring the dynamics of electrically oscillating DNA probes and their extension when aligned in an upright orientation provides comprehensive information on the complex formation of polymerase/DNA/dNTPs, and also on the enzymatic activity of the polymerase. The kinetic rate constants, affinities, Michaelis constant and turnover number which were determined on-chip were found to agree quantitatively with benchmark studies performed in solution (Table 2). This is significant since previous biosensor approaches have failed to reproduce solution values, most likely because of too high surface probe densities. While conventional immobilization schemes on streptavidin modified SPR sensors produce an inter-strand spacing of app. 2–6 nm^19^,^53^, the automated electrical desorption routines implemented in the DRX instrument used here enable an inter-strand spacing in excess of 30 nm, which helps to reduce steric interactions.

The fact that neither the enzyme nor the dNTPs need to be labelled for the current approach is of importance for the artefact-free analysis of incorporation rates, in particular with regard to applying the method for the development of single molecule sequencing and PCR techniques, where the performance of mutated polymerases and potentially chemically modified dNTPs needs to be compared to their native counterparts^3^. In the present study, the dye label was positioned at the 5’-end of the template strand (18 nt from the initial incorporation site) and was not found to impair polymerase binding or enzymatic activity.

It is straightforward to test many different measurement conditions in a single workflow utilizing the integrated microfluidics. Additionally, two methods are available to regenerate the chip surface: 1) rinsing with 5 *M* urea for protein denaturation, or 2) rinsing with NaOH for DNA denaturation. This permits the dissection of the different binding modes of Pol I(KF) to DNA and to elucidate how the binding affinity of the P-DNA interaction is modulated by the presence of cofactors. For example, in the absence of competing DNA, dNTPs, and Mg^2+^ in solution the binary P-DNA complex exhibits a previously unidentified stability (*k*_*off*_ = 3 × 10^−5^ *s*^−1^), which might prove useful for technical applications. The binding properties could further be characterized in temperature dependent measurements, which allowed the analysis of the enthalpic and entropic contributions of the bound state as well as of the activation barrier.

For dNTP binding, different affinities could be discriminated depending on whether the polymerase was located at the ss-ds fork (*K*_*D*_ = 0.6 μ*M*) or whether the polymerase was located on top of a just-polymerized double strand (*K*_*D*_ = 11 μ*M*). The primer elongation rate could be measured from both, the absolute increase in fluorescence and from the decreasing switching speed. The fluorescence increase seems better suited, however, because it is hardly affected by the commencing dissociation of the polymerase after it has reached the end of the template. In the switching speed measurement, on the contrary, effects of elongation and polymerase dissociation superimpose, resulting in an asymmetric transient downward pulse.

The switching speed measurement was found to be very sensitive to changes in the polymerase conformation. Hence, for the first time the finger-closing transition upon dNTP binding could be observed with a commercial biosensor and without the need for labelling.

Experiments with variable DNA template sequences and a large number of different polymerases or chemically modified dNTPs are beyond the scope of this paper, but seem straightforward utilizing the integrated autosampler and capabilities for parallel measurements. Thus, we believe the described method should be useful as a generic measurement modality for the comparative quantitative analysis of DNA as well as RNA polymerase synthesis processes.

## Methods

### Chip and DNA layer preparation

**Figure 7:**
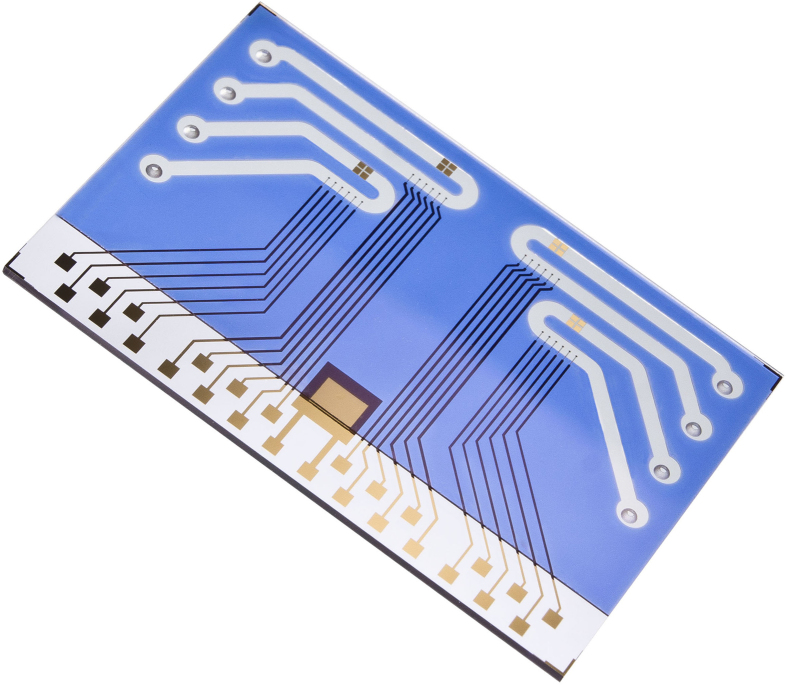
Chip with 4 flow channels and 24 microelectrodes.

Experiments were performed using “4 × 6” chips (Fig. 7) with “T54P36” surface modification (Dynamic Biosensors, Germany). The chips feature four separate flow channels with six gold work electrodes each (120 μm diameter) on glass substrates with a common transparent ITO counter electrode. Gold microelectrodes were modified with binary monolayers consisting of oligodeoxynucleotides and 6-Mercapto-1-hexanol (Sigma Aldrich). The sequence of the thiolated and Cy3 labelled 54-mer template strand was 5′ Cy3-ACC TTA GGC TGA TTA CTC GGT ATA GTC GAA TGC TGA GAA GTC GCA AGC TAC GTA-(CH_2_)_6_-SH 3′, the sequence of the complementary 36-mer oligodeoxynucleotide primer was 5′ TAC GTA GCT TGC GAC TTC TCA GCA TTC GAC TAT ACC 3′ (Metabion, Germany). The DNA density was adjusted by applying negative desorption voltages (e.g. −0.7 V vs. ITO) to obtain a low-density DNA layer with optimal switching properties (density estimation <10^10^ molecules/cm^2^). Prior to the chip being used for the first time, all primer DNA was stripped off with a pH 13 NaOH solution (100 mM, less than 1 s contact time) and new complementary primer DNA was hybridized (1 μM primer in Ca^2+^-buffer: 10 mM Tris pH 8.3, 40 mM KCl, 1.5 mM CaCl_2_) in order to yield a close to 100% hybridized template/primer DNA layer. This regeneration process was repeated throughout all experiments whenever any bound polymerase or elongated primer was needed to be removed and the initial layer of template/primer DNA reconstituted. Removal of only the polymerase but not the DNA primer was achieved by rinsing the surface with a buffered 5 M urea solution (1 min contact time).

Flow rates between 200 μl/min and 1000 μl/min were typically used during polymerase binding experiments, which was high enough to operate the sensor in the reaction-limited kinetics regime. An effect of the applied hydrodynamic flow on the DNA switching behaviour was not observed. Elongation and dissociation reactions were measured with flow rates between 5 μl/min and 20 μl/min. A Peltier element integrated in the sample holder enabled temperature controlled experiments. Tween (0.05%) was added to all buffers as surfactant.

### Biosensor setup and DNA switching potentials

A switchSENSE DRX-2400 instrument (Dynamic Biosensors, Germany) was used for actuation and analysis of the electrically switchable biosurface, the integrated time-correlating single-photon counter (TCSPC) setup for the switching dynamics measurement has been described elsewhere^40^. Prior to every time-resolved (TR) fluorescence measurement, the static fluorescence response to an applied voltage ramp was recorded in order to determine the switching potentials for the TR measurement, which were chosen close to the two ‘plateau regions’ of the fluorescence response where the DNA is completely lying or standing, respectively. The voltage amplitude was 0.8 V and the frequency of the applied square wave potential was 10 kHz, which ensured that the DNA had enough time to stand up and lie down completely before reversing the voltage.

### Fluorescence and Dynamic Response signals

From every TR measurement, two observables can be extracted: The absolute fluorescence signal at the negative switching potential (*F*_*up*_), indicating the position of the Cy3 dye above the Au surface when the DNA is standing, as well as the Dynamic Response signal of the upward motion (*DR*_*up*_), indicating the ‘switching speed’ of the DNA, an indicator for the total hydrodynamic friction of the DNA or the DNA-polymerase complex. The absolute fluorescence signal at the negative switching potential is calculated as the average fluorescence value of the last 5 μs when the DNA is standing, just before the potential is reversed again


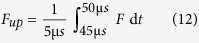


The Dynamic Response for the upward motion is calculated from the first 4 μs of the normalized fluorescence signal *F*_*norm*_, (Eq. 1). Photobleaching did not affect the measurements: because the fluorescence intensity is being normalized during the analysis of the dynamic response units, the switching speed does not dependent on the number of observed DNA strands (given that the signal is still high enough to be measured). Real-time elongation measurements were performed on a timescale much shorter than the typical photobleaching time constants (seconds vs. hours).

### DNA Polymerases and dNTPs

DNA Polymerase I Large (Klenow) Fragment (KF) and Klenow Fragment without exonuclease activity (3’ → 5’ exo^−^, with mutations D355A and E357A that abolish exonuclease activity) were obtained from New England Biolabs (Frankfurt a. M., Germany). The *Taq* DNA polymerase and deoxyribonucleotides (dNTPs) were obtained from Peqlab (Erlangen, Germany). The dNTPs were purchased either as ready-to-use dNTPs mix (10 mM) for elongation experiments or as individual dNTPs (100 mM) for the analysis of conformational changes.

### Association and dissociation measurements

The *Taq* binding experiments were performed in Mg^2+^ buffer (10 mM Tris pH 8.3, 40 mM KCl, 1.5 mM MgCl_2_). In the association measurements, 1 ml of *Taq* solution (concentrations from 1 mU/μl to 6 mU/μl) was injected with a flow rate of 200 μl/min for a 5 min association phase. Dissociation was measured for 1 h by rinsing 1200 μl with a flow rate of 20 μl/min over the chip. When measuring at temperatures below or above room temperature, the sample holder was precooled or -heated, respectively.

Due to the exonuclease activity of Pol I(KF), KF binding experiments were performed in Ca^2+^ buffer instead of Mg^2+^ buffer in order to prevent degradation of the DNA primer. The association of KF was measured via a 1.5 min injection of 1.5 ml of a 2 nM KF solution with a flow rate of 1000 μl/min. Dissociations were measured either in Ca^2+^ buffer (to suppress exonuclease activity when needed) or Mg^2+^ buffer mixed with dNTPs. The temperature in all experiments was 25 °C. Associations and dissociations were fitted with single- or bi-exponential functions.

### Elongation measurements

The elongation of *Taq* was measured at temperatures between 5 °C and 45 °C. The elongation reaction was conducted by first binding *Taq* in Mg^2+^ buffer without dNTPs to the template/primer DNA and then initiated by injection of a mix of all four dNTPs (100 μM) in Mg^2+^ buffer. The dNTP stock was diluted right before the experiment to prevent hydrolysis of the dNTPs. To determine the elongation rate, *F*_*up*_ was fitted with a single-exponential function. To obtain a higher sampling rate, the elongation at 45 °C was measured at constant negative potential, so that the DNA was standing throughout the whole elongation process and the fluorescence could be sampled continuously instead of only 5 μs out of 100 μs (see Fluorescence and Dynamic Response Signals).

The elongation of Pol I(KF) was measured at 25 °C and triggered by injecting a mix of all four dNTPs at concentrations between 10 nM and 100 μM. The elongation rate was determined by linear fitting of *F*_*up*_ (Michaelis-Menten kinetics).

### Analysis of conformational changes

For the analysis of conformational changes upon dNTP binding, 36 mer primer oligonucleotides terminated with a dideoxynucleotide were used. The sequence was 5′ TAC GTA GCT TGC GAC TTC TCA GCA TTC GAC TAT ACddC 3′. After binding the exonuclease deficient Pol I(KF-) to the template/primer DNA, Mg^2+^ buffer mixed with only one of the four dNTPs (10 nM–300 μM) was injected. After app. 30 s incubation time, five to seven individual TR measurements (20 s) were performed and the Dynamic Response of all measurements was averaged to yield one data point. During all measurements, dissociation of the polymerase from the template/primer DNA was prevented by keeping a sufficient background concentration of Pol I(KF).

### Control experiments with internally labelled template DNA

To investigate the effect on the dissociation rate of the polymerase by the end-labelling of the template oligonucleotide with a Cy3 dye, a template oligonucleotide with an internal labelling was used. The sequence was 5′ ACC TTA GGC TGA TTA CTC GG(T-Cy3) ATA GTC GAA TGC TGA GAA GTC GCA AGC TAC GTA-(CH_2_)_6_-SH 3′.

## Additional Information

**How to cite this article**: Langer, A. *et al.* Polymerase/DNA interactions and enzymatic activity: multi-parameter analysis with electro-switchable biosurfaces. *Sci. Rep.* **5**, 12066; doi: 10.1038/srep12066 (2015).

## Supplementary Material

Supporting Information

## Figures and Tables

**Table 1:** Thermodynamic energies of the *Taq*-DNA interaction in the absence of dNTPs (1 ↔ 2 in Fig. 1) determined by van’t Hoff and Eyring analyses from 5° to 55 °C.

		Transition state	Bound state
*ss*-*dsDNA*	Δ*H* (*kJ*·*mol*^−1^)	13.0 ± 2.1	24.9 ± 2.5
	Δ*S* (*J*·*mol*^−1^·*K*^−1^)	−62.8 ± 6.9	303 ± 9
	Δ*G*^25°C^ (*kJ*·*mol*^−1^)	31.7 ± 2.9	−65.5 ± 3.6
*dsDNA*	Δ*H* (*kJ*·*mol*^−1^)	10.0 ± 1.0	31.0 ± 1.7
	Δ*S* (*J*·*mol*^−1^·*K*^−1^)	−77.4 ± 3.2	302 ± 6
	Δ*G*^25°C^ (*kJ*·*mol*^−1^)	32.9 ± 1.4	−59.2 ± 2.3

**Table 2:** Pol I (KF) binding and enzymatic activity data measured using electro-switchable DNA layers in pH 8.3 buffer (10 mM Tris, 40 mM KCl) and comparison to solution measurements performed under comparable conditions.

		electro-switchable DNA layers		solution measurements	
P-DNA association	+1.5 mM Mg^2+^	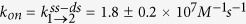		*k*_*on*_ = 1.2 × 10^7^ *M*^−1^ *s*^−1^(iv)	
P-DNA dissociation		*k*_*off*_	*K*_*D*_	*k*_*off*_	*K*_*D*_
	+1.5 mM Ca^2+^	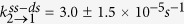	*K*_*D*,1↔2_ = 1.6 ± 0.8 *pM*	*n.a.*	*n.a.*
	+1.5 mM Mg^2+^	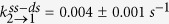	*K*_*D*,1↔2_ = 0.22 ± 0.05 *nM*	*n.a.*	*n.a.*
	+100 nM compet.DNA				
	+1.5 mM Mg^2+^	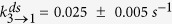	*K*_*D*,1↔3_ = 1.3 ± 0.4 *nM*	*n.a.*	*n.a.*
	+100 μM dNTPs				
	+1.5 mM Mg^2+^	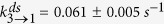	*K*_*D*,1↔3_ = 3.2 ± 0.6 *nM*	*k*_*off*_ = 0.06 *s*^−1^(iv)	*K*_*D*_ = 5–8 *nM*(iv)
	+100 nM compet.DNA				
	+100 μM dNTPs				
dNTP binding to P-DNA and incorporation rate	Dissociation after primer extension	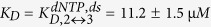 (i)		*K*_*D*_ = 6–17 μ*M*(iv)	
	Michaelis constant from elongation	*K*_*M*_ = 0.58 ± 0.20 μ*M*(ii)		*K*_*M*_ = 0.41 ± 0.17 μ*M*(v)	
	Use of dideoxy terminated primer	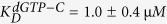 (iii)		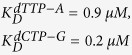 (vi)	
	Real-time elongation	*k*_*cat*_ = 2.0 *s*^−1^(ii)		*k*_*cat*_ = 2.4 *s*^−1^(iv)	

^(i)^determined from polymerase dissociation after elongation with 100 μM dNTPs in Mg^2+^-buffer.

^(ii)^determined from real-time elongation meas. (fluorescence increase of standing DNA) in Mg^2+^-buffer.

^(iii)^determined from conf. change upon fingers-closing.

^(iv)^chemical quench studies of primer extension using radiolabeling^13^,^47^.

^(v)^primer elongation gel shift assay^27^.

^(vi)^FRET study with a site-specifically labeled Pol I(KF)^11^.
