# Supplementary material for: Polymerase/DNA interactions and enzymatic activity: multi-parameter analysis with electro-switchable biosurfaces
Source: Sci Rep. 2015 Jul 15;5:12066. doi: 10.1038/srep12066 (PMC4502528; doi:10.1038/srep12066)
Supplement: Supporting Information [file srep12066-s1.pdf]

Polymerase/DNA interactions and enzymatic activity: multi-parameter analysis with electro-switchable biosurfaces

Andreas Langer[1,2], Michael Schräml[3,*], Ralf Strasser[1], Herwin Daub[1,2], Thomas W. Myers[4], Dieter Heindl[3], Ulrich Rant[1,*]

[1]Dynamic Biosensors GmbH, Lochhamer Str. 15, 82152 Martinsried, Germany

[2]Technische Universität München, 85748 Garching, Germany

[3]Roche Diagnostics GmbH, Nonnenwald 2, 82377 Penzberg, Germany

[4]Roche Molecular Systems Inc., 4300 Hacienda Dr Pleasanton, CA 94588, USA

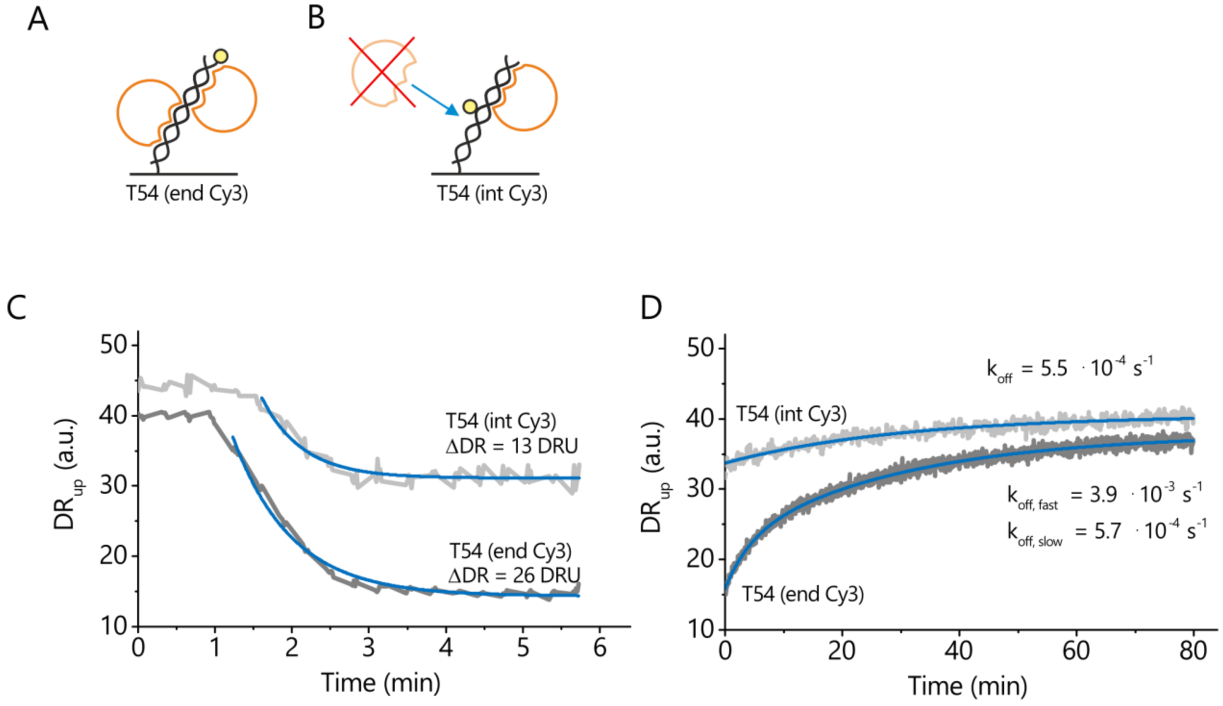

Supplementary Figure S1 | Effect of the fluorophore position on polymerae binding. *Taq* polymerase was bound to double-stranded 54mer DNA with varying positions of the Cy3 fluorophore: at the end (end Cy3, **panel A**) and internally (int Cy3, **panel B**). Absolute drops of the Dynamic Response in the association (panel **C**) and one respectively two time-constants in the dissociation (panel **D**) indicate binding of only one polymerase to the internally labeled DNA and two polymerases to the end-labeled DNA.

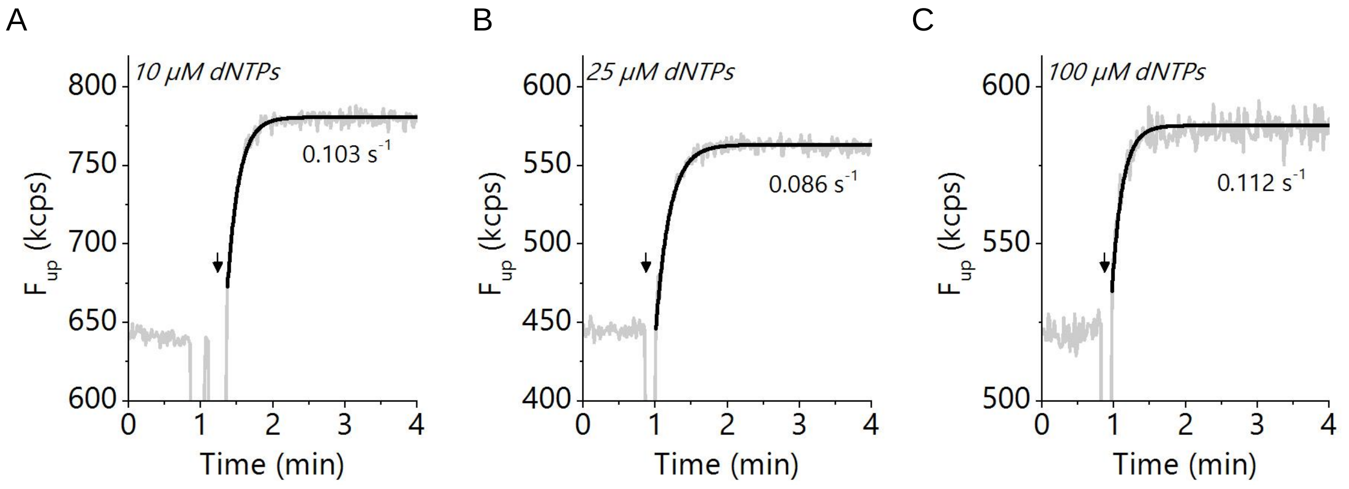

Supplementary Figure S2 | Elongation activity as a function of dNTP concentration. The elongation rate of *Taq* polymerase at 25°C was analyzed for dNTP concentrations of 10 µM (**A**), 25 µM (**B**) and 100 µM (**C**).
